# Advances in nanotechnology-based approaches for the treatment of head and neck squamous cell carcinoma

**DOI:** 10.1039/d4ra07193j

**Published:** 2024-12-09

**Authors:** Bicai Tang, Rui Huang, Wenjuan Ma

**Affiliations:** a State Key Laboratory of Oral Diseases, National Center for Stomatology, National Clinical Research Center for Oral Diseases, West China Hospital of Stomatology, Sichuan University Chengdu Sichuan 610041 China; b Sichuan Provincial Engineering Research Center of Oral Biomaterials Chengdu Sichuan 610041 China; c Department of Oral and Maxillofacial Surgery, West China Hospital of Stomatology, Sichuan University Chengdu 610041 China

## Abstract

Head and neck squamous cell carcinoma (HNSCC), one of the most common types of cancers occurring in the head and neck region, is often associated with high mortality rates due to its invasiveness and morbidity. The mainstream treatment methods in clinical settings, including surgery, chemotherapy, and radiotherapy, may cause poor overall survival rate and prognosis, with issues such as drug resistance, damage to adjacent healthy tissues, and potential recurrences. Other treatment approaches such as immunotherapy, photodynamic therapy (PDT), and photothermal therapy (PPT) also suffer from inefficient tumor targeting and suboptimal therapeutic outcomes. Early detection is vital for HNSCC patients, but it is always limited by insensitivity and confusing clinical manifestations. Hence, it is highly desirable to develop optimized therapeutic and diagnostic strategies. With the boom in nanomaterials, nanotechnology-conducted HNSCC therapy has attracted widespread attention. Nanoparticles (NPs) are distinguished by their unique morphology and superior physicochemical property, and some can exhibit direct antitumor activity, while others serve as promising candidates for drug delivery. In addition, NPs offer the potential for structural modification for drug delivery and tumor targeting, enabling specific delivery to tumor cells through conjugation with biomarker ligands and improving cargo biocompatibility. This work reviews current therapies and diagnosis methods for HNSCC, highlights the characteristics of the major NPs, surveys their uses and advantages in the treatment of HNSCC, and discusses the obstacles and prospects in clinical applications, aiming to enlighten future research directions for nanotechnology-based therapy for HNSCC.

## Abstract 2

Emerging nanoparticles (NPs), including biomembrane-based, polymeric, metallic, and non-metallic inorganic NPs, have offered powerful and promising approaches for the diagnosis and treatment of head and neck squamous cell carcinoma (HNSCC).

## Graphical abstract

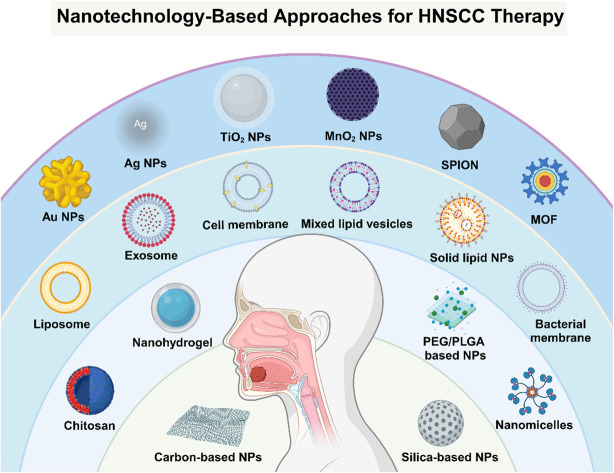

## 1. Introduction

Head and neck squamous cell carcinoma (HNSCC), which occurs in the mucosal epithelium of the oral cavity, pharynx and larynx, accounts for 90–95% of malignant tumors of the head and neck and ranks as the world's 6th common malignancy.^1,2^ The HNSCC prevalence rate in males is higher than that in females, and middle-aged to elderly men are the most vulnerable.^3^ The high incidence and poor prognosis of HNSCC impose a significant burden on healthcare systems and the economy. In 2018, the annual incidence of HNSCC was 890 000 with 450 000 deaths, which has been predicted to rise by approximately 30% in 2030 (*i.e.*, 1.08 million new cases annually) according to GLOBOCAN (an important global database of cancer statistics), demonstrating its rapidly increasing occurrence.^4^ In addition, a large proportion of patients with advanced HNSCC experience local and distant failure after treatment.^5^ Early-stage treatment of HNSCC shows the most favorable prognosis, which emphasizes the importance of early detection.^6^ However, as the pathological phenotype of HNSCC can be confused with other diseases, the early diagnosis rate of HNSCC is low, and the majority of patients are already in the advanced stage of diagnosis.^7^ Mainstream therapies for HNSCC include surgery in localized or early lesions, radiotherapy and chemotherapy for advanced or metastasized lesions, and immunotherapy; however, they still face non-negligible limitations.^8–11^ Damage to healthy tissues, unavoidable side effects, risk of recurrence, and drug resistance are the critical limitations of traditional clinical treatments that lead to the unsatisfactory prognosis of HNSCC. Despite recent progress in the therapeutic modalities for HNSCC, overall survival rates have not improved, and more alternative therapeutic approaches are highly desirable.^12,13^

Nowadays, the emergence of nanomedicine has provided new options for the treatment of various diseases. Owing to their biocompatibility, stability, controlled cargo release, targeting ability, easy preparation, and flexible modification, nanoparticles (NPs) have been widely studied for antitumor therapies.^14^ NPs possess optical, magnetic and electrical properties, making them suitable for establishing “smart” delivery systems that realize condition-sensitive drug release such as exogenous physical stimulations (*e.g.*, light, temperature and sound) or endogenous biological substances (*e.g.*, enzymes, reactive oxygen species (ROS), and ions).^14^ Different NPs can vary in material units of construction, morphological size, and surface properties, providing a wide range of selections for specific therapeutic or diagnostic purposes. Some NPs have shown great potential in various HNSCC therapies, which include biomembrane-based NPs, polymeric NPs, metallic NPs, and non-metallic inorganic NPs. In the review, we provide an overview of current treatment methods of HNSCC and discuss their limitations. We further highlight the intriguing characteristics of some major NPs and their advances for the treatment of HNSCC, which may propose novel therapeutic approaches for HNSCC in the future.

## 2. Risk factors, clinical manifestation, and pathological process of HNSCC

**Fig. 1:**
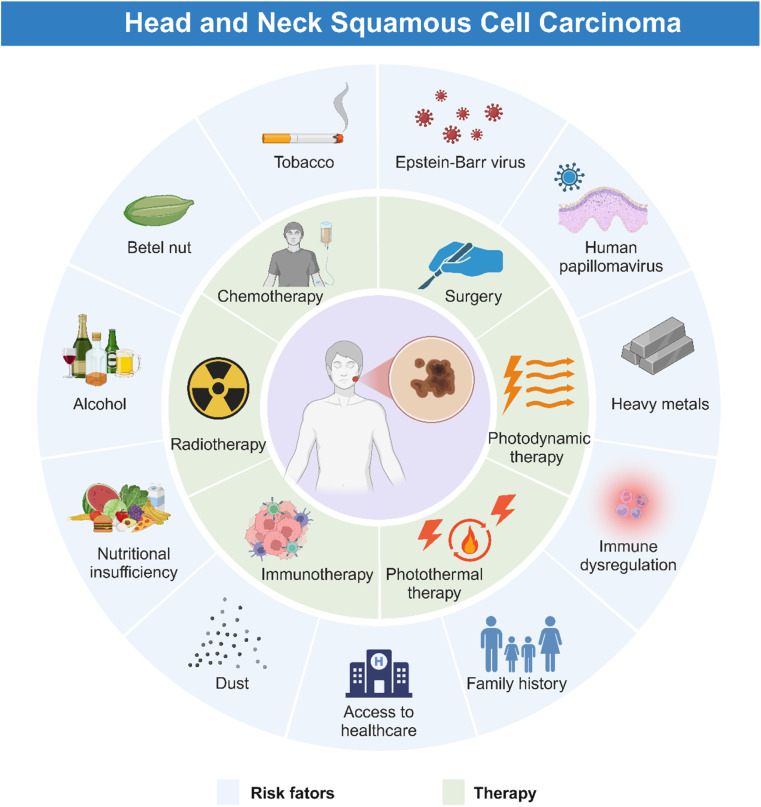
Risk factors and current therapy for HNSCC. The development of HNSCC is influenced by various risk factors, encompassing community, environmental and individual factors. Current therapies for HNSCC include surgery, chemotherapy, radiotherapy, immunotherapy, photodynamic therapy (PDT), and photothermal therapy (PTT).

HNSCC is associated with plenty of risk factors (Fig. 1), of which tobacco products are the most clearly identified.^15^ Harmful components in tobacco including polycyclic aromatic hydrocarbons (PAHs) and tobacco-specific nitrosamines (TSNAs) have been proved carcinogenic.^16^ In addition, alcohol induced HNSCC through the acetaldehyde metabolized by ethanol in the mouth.^17^ Consumption of areca nut products is another common reason for HNSCC, due to high concentration of alkaloids in the nut, which can disrupt the flora balance and then cause complex inflammation.^18^ Moreover, exposure to human papillomavirus (HPV), Epstein–Barr virus (EBV), poor oral hygiene, and heavy metals have been regarded as potential predisposing factors for HNSCC.^19^ Nutritional deficiency, especially plant foods and vitamin D, also contributes to the occurrence of HNSCC.^20^ Furthermore, HNSCC could occur at an early age because of the family history of some genetic alternations such as Xeroderma pigmentosum, Fanconi anemia, and Dyskeratosis congenital.^21^

**Fig. 2:**
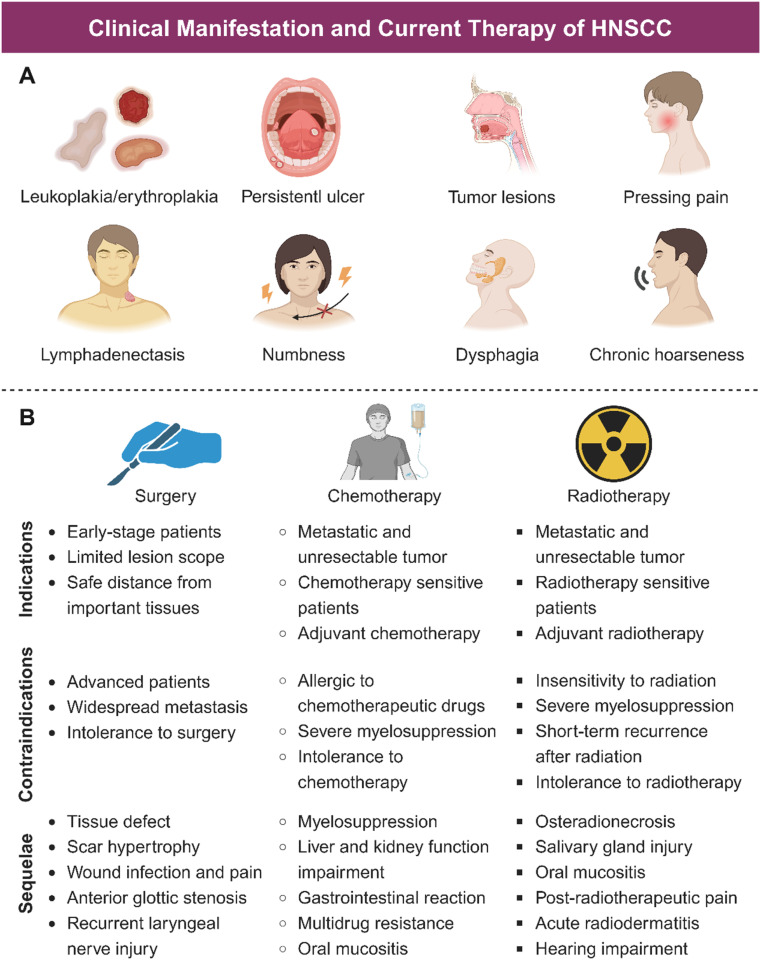
Clinical manifestations and current therapy of HNSCC. (A) Classic manifestation of HNSCC in the early stage mainly includes leukoplakia/erythroplakia and persistent ulcer. As the disease progresses, tumor mass would appear even accompanied with hemorrhage, pressing pain and lymphadenectasis. Some severe symptoms such as numbness, abnormal occlusion, difficulty in chewing or swallowing, difficulty in moving the tongue or temporomandibular joint, and chronic hoarseness, caused by tumor occupation or invasion, seriously impair the life quality of patients. (B) Mainstream HNSCC treatment methods represent surgery, chemotherapy and radiotherapy. Traditional therapies have limited scope of applications and non-negligible adverse effects, which has driven the search for alternative therapies.

HNSCC mainly occurs in oropharynx, oral cavity, laryngopharynx, hypopharynx, and larynx, manifesting similar symptoms.^22^ As illustrated in Fig. 1 and 2A, HNSCC in mucosa clinically presents as a well-defined lesion with white or red color.^23,24^ Ulceration with an irregular floor is regarded as the most typical symptom of HNSCC, which is hard upon palpation.^19,25^ Squamous cell carcinoma in the oropharynx and hypopharynx exhibits hidden symptoms at an early stage due to anatomical location, which usually indicate an advanced tumor progression when symptoms such as dysphagia, odynophagia and otalgia appear.^4^ For laryngeal squamous cell carcinoma, it deserves attention when patients exhibit symptoms of voice change and florid hoarseness that contributes to early diagnosis. If tumor lesions continue to growth, patients would suffer from dyspnea and airway obstruction and finally need tracheostomy to assist breathing.^4^ Moreover, other common symptoms such as persistent pain, tumor masses, halitosis, limited mouth opening, dyspnea, paresthesia, and bleeding may further lead to impaired sensation and motor function, causing great inconvenience to patients' lives. As for the most common kind of HNSCC, oral squamous cell carcinoma (OSCC), in addition to the typical symptoms, some oral potentially malignant disorders (OPMDs) such as oral leukoplakia, erythroplakia, submucosal fibrosis, and lichen planus are reported to be more likely to develop oral carcinomas.^26–28^ Moreover, lymphatic metastasis and hematogenous dissemination are both common manifestations for patients with advanced HNSCC.^29–31^ Histologically, HNSCC progresses in an orderly manner, beginning with epithelial cell hyperplasia, then dysplasia (mild, moderate, and severe), followed by preinvasive carcinoma, and finally invasive carcinoma.

At the genetic level, the occurrence and development of HNSCC have been shown to be related to the aberrant activation or up-regulation of oncogenic signaling pathways. The activation of the epidermal growth factor receptors (EGFR) pathway occurs most frequently in HNSCC, which greatly enhances the proliferation, metastasis and invasion of tumor cells and is associated with drug resistance and poor prognosis of HNSCC.^32,33^ Other oncogenic signaling pathways reported to be activated in HNSCC include Wnt/β-catenin, JAK/STAT, NOTCH, PI3K/AKT/mTOR, MET, and RAS/RAF/MAPK.^34–38^ The TP53/RB and p16/Cyclin D1/Rb pathways are the two most commonly seen aberrantly inactivated suppressor signaling pathways in OSCC that leads to uncontrolled cell proliferation and cell cycle, and ultimately dysplasia, significantly contributing to the progression of HNSCC.^39,40^ Additionally, the dysregulated tumor microenvironment (TME) caused by immune suppression, hypoxia, abnormal stromal components, and dysbacteriosis has also been recognized to play an essential role in the pathological process of HNSCC.^41–43^

## 3. Current therapy for HNSCC

Surgery is the first choice to treat HNSCC patients at the early stage. Small tumors can usually be resected transorally, while large tumors or those with posterior extension to other tissues are hard to be completely removed, which could even increase the possibility of regional recurrences and affect long-term survival rates.^44^ As an aggressively intrusive therapy, surgery has unavoidable consequences, leading to facial deformity and oral physiological dysfunction, including speech impairment, dysphagia, and chewing impairment, which consequently damage the post-treatment life quality of HNSCC patients.^45–48^

For patients with metastases, unresectable tumors, and advanced stage, radiotherapy and chemotherapy are the main therapeutic methods. Radiotherapy induces cell apoptosis through DNA damage caused by high-energy ionizing radiation or production of ROS, which is less affected by appearance and tissue depth.^49^ Nevertheless, radiotherapy has limitations in clinic applications. On the one hand, radiation-related resistance of tumor cells reduces efficacy; on the other hand, radiation sequelae such as osteoradionecrosis (ORN), acute radiodermatitis (ARD), salivary gland injury, and mucositis, could lead to xerostomia, severe caries, pain, and dysgeusia (Fig. 2A), which seriously affect patients' daily life.^50^ Chemotherapy inhibits tumor growth by blocking cell cycle progression with chemotherapeutic agents. However, serious side effects and drug resistance are the major hurdles of poor prognosis of many patients treated with chemotherapy.^51^ Other problems including low bioavailability, non-specific biological distribution, poor tumor aggregation, and undesirable anticancer efficiency also limited the application of chemotherapy.^52^ Indications, contraindications, and sequelae of mainstream HNSCC therapy are shown in Fig. 2B.

In addition to the above-mentioned classic treatment methods, other treatment methods include immunotherapy, photodynamic therapy (PDT), photothermal therapy (PTT), sonodynamic therapy (SDT), and bacterial therapy (Fig. 1). These emerging therapeutic approaches have demonstrated effectiveness to some degree, but they could not fully address the challenges posed by traditional therapies, such as the tolerance of tumor and considerable adverse effects. Clinically, the limitations of these treatments are also reflected in the fact that they are not suitable for all patients. Therefore, alternative therapy for HNSCC is necessary.

## 4. Application of NP-based therapy for HNSCC

With the advancement of nanotechnology, emerging NPs have been employed in order to improve the therapeutic effects of classic treatment methods. NPs are materials typically of size ranging between 100 and 1000 nm, possessing unique mechanical and physicochemical properties. Some NPs can directly perform own therapeutic functions. Others have structures to preserve and precisely transport functional cargos, acting as vehicles for the drug delivery system. For the advantages of diversiform structural editability, NPs could be endowed with the multimode therapeutic abilities such as the combination of chemotherapy and immunotherapy.^53–55^ Therefore, the application of NPs may increase the accumulation and bioavailability of therapeutic agents in the original and metastatic tumor sites when compared to traditional HNSCC therapy, thus shortening treatment cycle, enhancing organ targeting, and improving therapeutic outcomes.^56^ Moreover, NPs can be easily functionalized in various ways to optimize their targeting ability and stability, thus achieving maximum effectiveness with less side effects on health tissues. The most common NPs used in HNSCC include biomembrane-based NPs, polymeric NPs, metallic NPs, and non-metallic inorganic NPs.

### 4.1 Biomembrane-based NPs

**Fig. 3:**
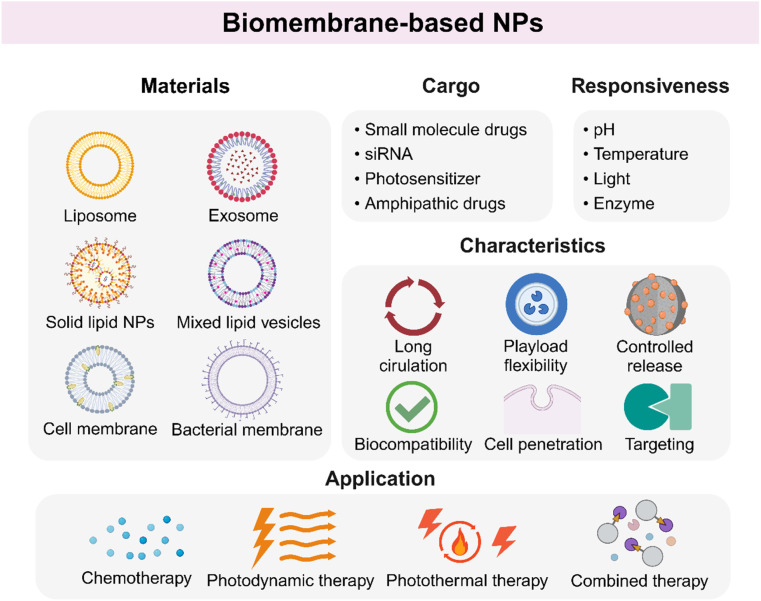
Biomembrane-based NPs differ from other NPs for unique phospholipid bilayer structures with low immunogenicity and biodegradability. Entrapping different types of cargos, vesicles are able to achieve multiple treatment modalities for HNSCC. In addition, biomembrane-based NPs are often endowed with responsiveness to different conditions owing to their easily modified surface.

Biomembrane-based NPs refer to nanomaterials with a phospholipid bilayer structure, which are usually classified into two categories: artificial synthesis and natural membrane extraction.^57^ Artificial biomembrane nanostructures are mainly lipid-based NPs, while natural biomembrane nanostructures are derived from various sources including extracellular vesicles (EVs), cell membranes, and bacteria.^58,59^ Biomembrane-based NPs are an emerging category of NPs used for drug delivery on account of high drug-loading capacity, biocompatibility, and biodegradability. In addition, surface modification endows these materials with precise control of antitumor drugs (Fig. 3).

#### 4.1.1 Lipid-based NPs

Lipid-based NPs (LNPs) mainly include liposomes, solid lipid NPs (SLNs), hybrid lipid-polymer NPs, nanostructured lipid carriers (NLCs), and nonlamellar lipid NPs. LNPs have effectively exhibited the ability to increase the selectivity and solubility of insoluble anti-cancer drugs, and then reduced the cytotoxicity and toxic side effects, offering a high-efficiency delivery system with prolonged and controlled release of agents.^60^

Among them, liposomes are the most widely studied vesicle NPs. Liposomes are spherical lipid vesicles with a phospholipid bilayer structure, which are one of the first nanosized drug delivery systems.^61^ Liposomes could enhance the antitumor efficacy of chemotherapeutic agents with reduced side effects. Moreover, liposomes are able to entrap both hydrophobic and hydrophilic drugs due to the unique amphiphilic properties of phospholipids, hence improving the solubility, stability, and enhanced permeability and retention (EPR) effect of the loaded core drugs. El-Hamid *et al.* found that liposome-decorated doxorubicin (DOX) called Doxil showed a greater apoptotic effect on cells than free DOX with an increased level of C-Myc mRNA inhibition.^62^ Additionally, liposomes have been decorated with specific ligands to recognize and conjugate to receptors on cells, such as folate receptor (FR), in order to extend circulation time, increase tumor growth inhibition, and improve lifespan.^63^ Zheng and co-workers developed an injectable resveratrol (RSV) and EGFR targeting ligand GE11-conjugated liposome (RSV-GL), which significantly enhanced the cytotoxic effect on HNSCC cells compared to the non-targeted NPs.^64^ Another widely acceptable modification of liposomes is the condition-responsive drug release ability when exposed to physical and biological stimulations, or called stimuli-responsive liposomes. Furthermore, the application of liposome-based NPs for noninvasive bioimaging and treatment for HNSCC has been proved feasible. Wei *et al.* successfully integrated evodiamine (EVO) and photosensitizer indocyanine green (ICG) into a liposome-based nanoplatform, aiming to realize precise FI (fluorescence imaging) and PET-CT imaging-guided chemo/chemodynamic/photodynamic tri-modal therapy.^65^ The idea of using liposome-based NPs for the combination of noninvasive bioimaging and precise tumor killing is brand new. The liposome-based NPs not only serve as a highway to tumor tissues, but also provide condition-guided tumor treatment, especially for those who are old, have poor physical condition and are unable to withstand the side effects of treatment, which is of great significance.

In addition to liposomes, the emergence of SLNs represents the innovations in lipid-based nanotechnology.^66^ The solid lipid structure endows SLN with higher stability, greater loading capacity and bioavailability, and low toxicity.^67^ Li and colleagues used SLN to load andrographolide (ADG) named ADG-SLN that exhibited superior inhibitory activities against HNSCC cells over free ADG due to the improved efficiency of intracellular absorption.^68^ Although LNPs have exhibited therapeutic potential against HNSCC, the application of LNPs suffers from the problem of liver aggregation. Therefore, some researchers used an *in vivo* high-throughput assay to test the nucleic acid (mRNA vaccines) delivery function of 94 chemically distinct LNPs. They finally identified the greatest HNSCC-targeting LNP and called it LNP^HNSCC^, which reduced the off-target delivery of functional mRNA.^69^ The screening technique used in LNPs, named DNA barcoding, inspires future approaches to find appropriate LNPs for different solid tumors.

#### 4.1.2 Exosomes

EVs are membrane nanostructures generated from cells, containing various biomolecules and biological characteristics of the source cells, which have been declared very important in intercellular communication.^70^ EVs have been recognized as promising natural drug delivery systems for unique membrane structures and biological regulatory capacity.^71,72^ EVs are typically divided into three types according to biogenesis, composition and size: exosomes (EXOs), microvesicles and apoptotic bodies.^73^ Among them, the large-scale isolation and production of EXOs have been widely reported, making EXOs the most frequently used EVs for disease diagnosis and treatment.^74,75^ EXOs are a type of EVs originating from the late endosome with a size of 40–160 nm and composed of an internal aqueous medium and a lipid membrane containing an abundance of specific biomolecules.^76^ Bio-originated EXOs are widely deemed with higher biocompatibility, better cell and tissue penetration abilities, and lower immunogenicity and toxicity.^77^ Being crucial mediators of the intercellular communication, EXOs serve as promising vehicles for steady and effective delivery of biomolecules, especially naked nucleic acids.^78^ The applications of EXOs in HNSCC mainly included the studies of how tumor cell-derived EXOs influenced the growth and detection of HNSCC and the EXO-based therapeutic strategies for OSCC. For example, Sayyed *et al.* created miRNA-155 inhibitor-laden EXOs to reverse the chemoresistance of cisplatin in OSCC, offering an alternative therapy for the refractory oral cancer patients.^79^ Wang and colleagues employed EXOs to load transient receptor potential polycystic 2 (TRPP2)-siRNA, significantly reducing the epithelial–mesenchymal transition (EMT) in FaDu cells (derived from human pharyngeal squamous cell carcinoma), which was important for the invasion and metastasis of HNSCC.^80^ In a combinational treatment strategy, EXO-based NPs encapsulated a photosensitizer (Indocyanine green) and a tyrosine kinase inhibitor (Gefitinib) called IG@EXOs that was proved effective against OSCC *via* the synergistic PTT and targeted therapy.^81^ Previous studies have shown that EXOs are easy to be surface modified to optimize their performance.^82^ Qiu and co-workers developed a transfected mesenchymal stromal cell (MSC)-derived EXO vector for OSCC to deliver cabazitaxel (CTX) and tumor necrosis factor-related apoptosis-inducing ligand (TRAIL) combinations. The MSC-EXO exhibited excellent drug-loading and controlled-release ability, possessing a satisfying synergistic effect on tumor inhibition *in vitro* and *in vivo*.^83^ Surprisingly, the potential of EXOs as stimulus-activated drug delivery systems has also been explored. Zhang *et al.* employed DOX-conjugated milk-EXOs to encapsulate anthracene endoperoxide derivative (EPT1) and chlorin e6 (Ce6), conducting a pH/light-responsive vesicle system (Exo@DOX-EPT1 NPs) for OSCC treatment. *In vitro* and *in vivo* experiments supported the antitumor capability of Exo@DOX-EPT1 NPs with controlled drug-release and enhanced biocompatibility, which was proved as an effective treatment of OSCC.^84^

#### 4.1.3 Cell membrane-based NPs

As a natural multifunctional biomaterial, the cell membrane is gradually involved in the mass preparation of biomimetic vehicles. Based on the satisfying natural properties of cell membranes and developed surface modification forms, cell membrane-derived biomimetic vehicles are attracting much attention for excellent targeting ability, specific aggregation in injured cells, prolonged circulation time, low immunogenicity, and enhanced biocompatibility.^85^ Besides, cell membrane-based NPs have a tendency to cling to homologous cells, or called homing ability, allowing the possibility of personalized oncotherapy.^86,87^ Li *et al.* constructed macrophage-membrane-camouflaged chitosan NPs (CAs) to achieve the synergistic delivery of the miRNA-144/451a cluster. The system could protect miRNAs from RNase-A induced degradation and significantly reduce the cell viability, migration and invasion of HNSCC, providing a new idea for the application of gene co-transfection in oncotherapy.^88^ Besides, considering the relationship between HNSCC and various maxillofacial tissues, some hybrid cell membranes of multiple cell origins have been developed as functionalized biomimetic NPs. For example, Chen *et al.* prepared a hybrid membrane of red blood cell (RBC) and HNSCC cell (WSU-HN6) membranes and modified it with a bone-targeting polypeptide (Asp 8) to obtain a bionic shell of hypoxia-activated chemotherapeutic drug tirapazamine (TPZ) and photosensitizer IR780-loaded hyperbranched polymeric NPs. This type of NP based on hybrid membrane vesicles successfully achieved immune escape of kernel drugs and obtained the combined effect of jaw-targeting, PTT and hypoxia-activated chemotherapy.^89^ Although there are few studies on the applications of cell membrane-based NPs for HNSCC treatment, the good performances of these NPs in various tumor-related fields such as immune regulation, drug delivery, tissue targeting, genetic engineering, bioimaging, and combination therapy have been widely reported.^90–93^ Therefore, with the deepening of research, the importance of cell membrane-based NPs for future HNSCC therapy is self-evident.

#### 4.1.4 Bacteria-derived NPs

Bacteria-derived NPs are an important category of biomembrane-based NPs, which have been widely developed as tumor vaccines for immune activation function.^94,95^ As for the application of bacteria-derived NPs in HNSCC therapy, the effect of oral microbiota on inducing the onset and progression of OSCC has been widely recognized, especially *Porphyromonas gingivalis* (*Pg*), *Fusobacterium nucleatum* and *Streptococcus*, which has greatly promoted the application of bacteria-derived NPs for OSCC treatment.^96,97^ Among them, *Pg* could be identified as an ideal candidate for bacterial therapy for abundant pathogen-associated molecular patterns and protoporphyrin IX (PpIX), which promotes immune activation and photosensitivity.^98–100^ As one of the most ubiquitous components of ecosystem, bacteria could be easily obtained and retained in various tissues including tumor sites.^101^ Therefore, Shi *et al.* reported a bacterial nanomedicine (nm*Pg*) fabricated from *Pg* that exhibited powerful PDT and antitumor immune effects for OSCC treatment. Additionally, DOX was further loaded into nm*Pg* to enhance the tumoricidal performance. The nm*Pg*/DOX complex possessed superior drug-loading capacity and reliable stability, leading to synergistic antitumor ability of photodynamic-immunotherapy and chemotherapy.^102^ Moreover, *Pg*-derived membrane nanostructures contain both natural immunostimulatory effects and loading capacity, which are being developed into a brand new bacterial adjuvant.^103,104^ Chen and co-workers extracted double-layered membrane vesicles (DMVs) from attenuated *Pg* as a biomimetic vehicle for the delivery of photosensitizer IR780 and stabilizer poly(β-amino) ester (PBAE) called PBAE/IR780@DMV. PBAE/IR780@DMV possessed PTT-PDT synergetic therapeutic effects and DMV-stimulated immune response, resulting in robust antitumor activities for OSCC.^105^

Briefly, biomembrane-based NPs have attracted much attention for inherent excellent characteristics, becoming new selections for oncotherapy. The enthusiasm to explore the application prospects of biomembrane-based NPs is still growing, and more details are shown in Table 1 and Fig. 3.

**Table 1:** Biomembrane-based NPs applied for the treatment of head and neck squamous cell carcinoma

	Nanoparticles	Matrix	Agents	Biological applications	Ref.	Advantages
Lipid-based NPs	DOXiL	Liposome	Doxorubicin (DOX)	Chemotherapy	62	High encapsulation efficacy, low toxicity, and controlled-release property
	RSV-GL	Liposome	Resveratrol (RSV), EGFR targeting ligand GE11	Chemotherapy	64	
	EI@Lipo	Liposome	Evodiamine (EVO), indocyanine green (ICG)	Imaging, chemo/chemodynamic/photodynamic tri-modal therapy	65	
	ADG-SLN	Solid lipid NPs	Andrographolide (ADG)	Chemotherapy	68	
Exosome	MiRNA-155 inhibitor-laden EXO	Exosome	MiRNA-155 inhibitor	Targeted therapy	77	High biocompatibility, cell-penetration ability, and low immunogenicity
	EXOs/TRPP2 siRNA complexes	Exosome	Transient receptor potential polycystic 2 (TRPP2)-siRNA	Targeted therapy	80	
	IG@EXOs	Exosome	ICG, gefitinib	Phototherapy, targeted therapy	81	
	MSCT-EXO/CTX	Exosome	Cabazitaxel, tumor necrosis factor-related apoptosis-inducing ligand (TRAIL)	Chemotherapy, targeted therapy	83	
	Exo@DOX-EPT1	Exosome	DOX, endoperoxides derivative (EPT1), chlorin e6 (Ce6)	Chemotherapy, photo thermal therapy	84	
Cell membrane-based NPs	MEXO/CA-miR-451a	Macrophage membrane-camouflaged chitosan NPs (CAs)	MiRNA-144/451a cluster	Targeted therapy	106	Specific-targeting, high biocompatibility, prolonged circulation time, and low immunogenicity
	Asp8[H40-TPZ/IR780@(RBC-H)] NPs	Asp8-decorated RBC and WSU-HN6 cell hybrid membrane	Tirapazamine (TPZ) and photosensitizer IR780-loaded hyperbranched polymeric NPs	Targeted therapy, PTT, hypoxia-activated chemotherapy	89	
Bacteria-derived NPs	nm*Pg*/DOX	*Porphyromonas gingivalis* (*Pg*)-derived bacterial nanomedicine	DOX	Photodynamic-immunotherapy, chemotherapy, bacterial therapy	102	Immune activation, photosensitivity, and low-cost
	PBAE/IR780@DMV	*Pg*-derived double-layered membrane vesicles	IR780, poly(β-amino) ester (PBAE)	Photo thermal therapy, photodynamic therapy, immunotherapy, bacterial therapy	105	

### 4.2 Polymeric NPs

**Fig. 4:**
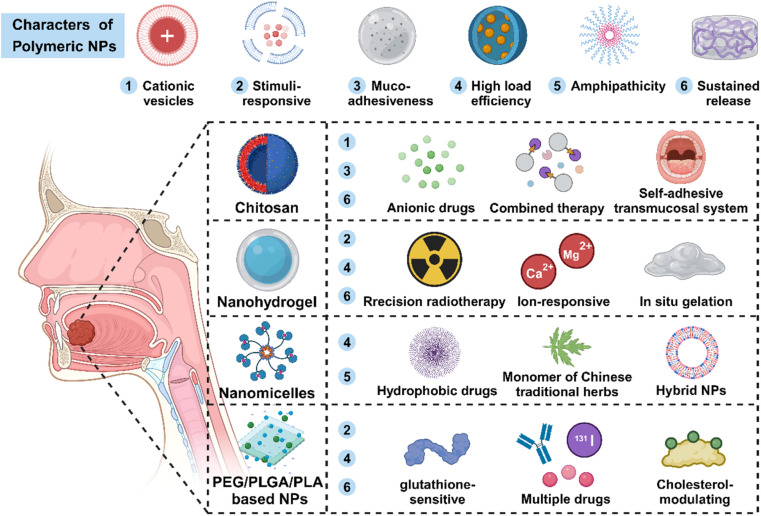
Polymeric NPs (PNPs) are regarded as ideal drug-delivery candidates for abundant advantages. Based on the properties of each NP, polymeric NPs are applied to load various drugs or are developed into special preparations such as slow-release gels or mucosal patches. Moreover, the flexible cross-linked structure gives them more opportunities for modification.

Polymeric NPs (PNPs) with flexible long-chain structure present significant advantages such as excellent stability and dispersibility, high cargo loading capacity, and ideal biocompatibility. PNPs have shown great promise as attractive platforms for oncotherapy (Fig. 4).^107^

#### 4.2.1 Chitosan

Chitosan is a linear polysaccharide biotechnological derivative of chitin. Chitosan-based NPs have gained great interest for their high drug-entrapment efficiencies, negligible toxicity, great biocompatibility, and prolonged circulation time.^108–111^ The cationic characteristics of chitosan have made it a popular choice to improve the delivery efficiency of anionic drugs and genes.^112^ Hariharan *et al.* designed an erlotinib (ERB, tyrosine kinase inhibitor)-laden liposome coated with the cationic polymer chitosan (CS) called CS-ERB Lipo for HNSCC treatment. The CS-ERB Lipo accomplished sustained-release up to 36 h from gel, resulting in high-efficacy uptake, mucous interaction ability, and potent antitumor activity.^113^ Moreover, chitosan NPs have become potential candidates for PDT. Yang and colleagues incorporated *N*-succinyl chitosan into the folic-acid-modified chitosan NPs to prepare fSCN NPs as 5-ALA (a widely studied phototherapy agent) carriers. The fSCNA NPs with high load capacity, stability, and cellular uptake, significantly enhancing the accumulation of photosensitive fluorophore protoporphyrin IX (PpIX), were regarded as excellent vectors for 5-ALA.^114^ Based on chitosan, a mitochondrial-targeting drug co-delivery system of 5-ALA-induced PpIX photosensitizer and mitochondrial glioblastoma-amplified sequence (GBAS) gene was successfully created for combined PDT and gene therapy, which exhibited great dispersion, stability, hypotoxicity and superior mitochondrially targeted killing effect on HNSCC.^115^

For hydrophilia, chitosan is an ideal raw material for biocompatible and biodegradable hydrogels. Chitosan exhibits excellent mucoadhesiveness, making it particularly suitable for mucosa drug administration in HNSCC.^116^ Goldberg *et al.* encapsulated cisplatin-loaded chitosan NPs into a chitosan sponge matrix to prepare PRV111, a self-adhesive transmucosal system. The system could leave drug-carrying NPs at the objective site, avoiding the inactivation of cisplatin. In a significant step, a phase I/II clinical trial reported that the chitosan-based nanomedicine performed well in patients with early-stage OSCC without severe adverse events.^117^

#### 4.2.2 Nanohydrogel

Nanohydrogel is the three-dimensional polymeric network considered as a strong delivery vector candidate for high payload capacity, biocompatibility, flexibility, and mechanical stability.^118^ Nowadays, condition-responsive nanohydrogels have been widely studied, as the loading and releasing of the core drugs could be precisely regulated by environmental stimulations.^119^ For example, an injectable, porous and temperature-sensitive hydrogel was developed to deliver astragalus polysaccharide (APS). The results indicated that the composite hydrogel could slowly release APS and greatly suppress the proliferation and migration of CAL-27 cells.^118^ In addition, to optimize the biodistribution of radiosensitizer poly(vinyl pyrrolidone)-modified tantalum NPs (Ta@PVP NPs), TME-responsive sodium alginate (DAA) *in situ* hydrogels were employed for precision radiotherapy. These ion-responsive Ta@PVP-DAA hydrogels are sensitive to the high Ca^2+^/Mg^2+^ environment in solid tumors and show effective HNSCC inhibition without any damage to the surrounding tissue under photothermal-assisted radiotherapy.^120^ Another study reported a nano-DOX-indocyanine green matrix metalloproteinase (MMP)-responsive hydrogel called NDIMH to combine chemotherapy and PTT, which achieved superior antitumor efficacy, acceptable biosafety and improved drug retention at the tumor site.^121^ To further improve the immunotherapy effectiveness for HNSCC, Wu *et al.* developed a kind of composite nanohydrogel based on a PLGA-PEG-PLGA framework, which was used to deliver both immune activator imiquimod-loaded CaCO3 NPs (RC) and cancer cell membrane (CCM)-decorated mesoporous silica NPs. The silica NPs contained paclitaxel and a proteolysis-targeting chimera (PROTAC) for BMI1 that promoted the tumor progression. Hence the injectable composite nanohydrogel has been employed as a reservoir for the complicated particle system and adhered to tumors.^122^ Nanohydrogels have also been reported to serve as dressings to prevent and treat ARD for post-radiotherapy HNSCC patients.^123^ Therefore, the application potential of nanohydrogels is far greater than we thought, which could act as versatile nanoplatforms with precise design.

#### 4.2.3 Nanomicelles

Polymeric nanomicelles (PNMs), one of a stable category of nanomicelles, are prepared from amphiphilic polymers and contain a hydrophobic inner core and a hydrophilic outer shell. PNMs stand out for good stability and biocompatibility, which tend to accumulate in the targeted site with enhanced therapeutic effect and reduced toxicity on noncancer tissues. Moreover, the characteristics of PNMs can be further changed by modification of the outer shell.

For example, Ma *et al.* prepared a hybrid cationic nanomicelle named DMP to load a plasmid encoding the apoptosis-inducing *BimS* gene, which possess wonderful antitumor properties, appropriate biocompatibility and ideal biosecurity.^124^ In another work, a complex consists of cationic micelles and siSTAT3 + siTGF-β was proved to have antitumor effects and bone invasion inhibition in an HNSCC mouse mandibular invasion model.^125^ Additionally, Tao *et al.* encapsulated hypoxia regulator RES and photodynamic agent Ce6 in GE11-decorated micelles. The micelles were admired for high cellular internalization, facilitating the penetration of drugs and increasing the synergistic antitumor effects of RES and Ce6.^126^ PNMs could also be developed as pH-responsive drug carriers to realize precise tumor release. Zhu and co-workers fabricated pH-sensitive amphiphilic block copolymer iPDPA to entrap oxaliplatin and dasatinib (a type of proto-oncogene Src inhibitors) named PDO NPs. The PDO NPs greatly activated antitumor immunity and promoted chemotherapy in the immunosuppressive TME, exhibiting expectable application prospects for HNSCC.^127^

#### 4.2.4 PEG/PLGA/PLA-based NPs

Polyethylene glycol (PEG) is the most widely used polymer in drug delivery systems due to tunable properties and safety. PEG-coated surface could protect various NPs from aggregation, opsonization, and phagocytosis, thereby prolonging the circulation time and decreasing immunogenicity without impact on activity. Zhang's group developed cholesterol-modulating nanoplatform PEG-terbinafine-Y8 NPs (PYT NPs), which showed great promise in stimulating cholesterol-modulating immunity when combined with photo-immunotherapy.^128^ In addition, PEG-based NPs have exhibited excellent tumor-targeting capability after modification. Fan *et al.* designed glutathione (GSH) and oleic acid (FA)-sensitive PEG NPs loaded with paclitaxel (PTX) called FA-NPs. This targeted drug delivery system realized sustained PTX release triggered by GSH response, leading to remarkable tumor inhibition *in vivo* with reduced adverse reactions.^129^ Moreover, the flexible size of PEG NPs allows them to encapsulate larger cores such as liposomes. A type of pH-sensitive and self-destructive PEG NPs act as shells of liposomes and SLN to load irinotecan and miRNA-200, successfully enhancing the tumor-specific accumulation of drugs.^130^

Furthermore, PEG could be applied to combine with poly(lactic-*co*-glycolic acid) (PLGA), constructing a brand new type of amphiphilic block co-polymer nanomaterials called PEG-PLGA NPs, which help modify the characteristics of PLGA NPs and obtain long-term therapeutic effects. In Chen's study, the antitumor agent all-trans retinoic acid (ATRA) was loaded into a PEG-PLGA complex and further modified with an anti-PD-L1 antibody. ATRA-PLGA-PEG-PD-L1 NPs possessed low immunogenicity, particularly targeting dysplastic and squamous carcinoma cells.^131^ Poly(lactic acid) (PLA) is another polymer widely used for the preparation of safe and effective delivery systems. Compared to PLGA, the hydrophobic side chain in the structure renders PLA with a lower water uptake property and slower degradation.^132^ Based on the excellent physicochemical property of PEG and PLA, Wu and co-workers synthesized tumor-targeting PEG-PLA NPs to deliver cetuximab, 5-FU, and radionuclide iodine-131 in a xenograft mouse model. This type of NPs exhibited prolonged circulation and tended to aggregate in the tumor site, hence exhibiting satisfying antitumor capacity.^133^ Coincidentally, Elsaddy *et al.* employed a PEG-PLA-PEG tri-block copolymer as a delivery vehicle to encapsulate different drugs including monoclonal antibody and chemotherapeutic agents for intralesional injection. Using the drug delivery system greatly increased the load capacity of hydrophobic drugs and prolonged the blood circulation time.^134^

As Table 2 and Fig. 4 show, polymeric NPs serving as one of the most vital types of drug delivery vehicles have exhibited growing potential in oncotherapy. These composite NPs that self-assembled by multiple molecules own more abundant properties than the single synthetic NPs, which greatly promote the process of multifunctional nanomaterials playing an active role in the field of biomedicine.

**Table 2:** Polymeric NPs applied for the treatment of head and neck squamous cell carcinoma

	Nanoparticles	Matrix	Agents	Biological applications	Ref.	Advantages
Chitosan-based NPs	CS-ERB Lipo	Chitosan-coated liposome	Erlotinib	Targeted therapy	113	High encapsulation efficacy, biocompatibility, hydrophilia, mucoadhesiveness, and prolonged circulation time
	fSCNA	Folic-acid-modified chitosan	5-Aminolevulinic acid (ALA)	Photodynamic therapy	114	
	CS-ALA-shGBAS	Chitosan	5-ALA-induced PpIX photosensitizer, mitochondrial genes glioblastoma-amplified sequence (GBAS)	Photodynamic therapy, gene therapy	115	
	PRV111	Chitosan sponge matrix	Cisplatin-loaded chitosan NPs	Chemotherapy	117	
Nanohydrogel	MTNs-APS@HA-PNIPAAM hydrogels	Composite hydrogel	Astragalus polysaccharide (APS)	Chemotherapy	118	High payload capacity, biocompatibility, structure-flexibility, and mechanical stability
	Ta@PVP-DAA hydrogels	Hydrogel	Poly (vinyl pyrrolidone)-modified tantalum nanoparticles (Ta@PVP)	Photothermal-assisted radiotherapy	120	
	NDIMH	Hydrogel	Doxorubicin (DOX), indocyanine green (ICG)	Chemotherapy, phototherapy	121	
	Nanocomposite hydrogel	Hydrogel based on PLGA-PEG-PLGA framework	Imiquimod encapsulated CaCO3 NPs (RC), cancer cell membrane (CCM)-coated mesoporous silica NPs with PROTAC for BMI1 and paclitaxel	Immunotherapy, chemotherapy	122	
Nanomicelles	DMP/phBimS	DOTAP-mPEG-PCL (DMP) micelles	BimS gene	Gene therapy	124	High stability, biocompatibility, tissue-targeting, and few side effect
	DMP/siSTAT3 + siTGF-β	DOTAP-mPEG-PCL (DMP) micelles	siSTAT3, siTGF-β	Gene therapy	125	
	RC-GMN	Ge11 modified micelles	Resveratrol (RSV), chlorine e6 (Ce6)	Chemotherapy, photodynamic therapy	126	
	PDO NPs	Amphiphilic block copolymer iPDPA	Dasatinib, oxaliplatin	Immunotherapy, chemotherapy	127	
PEG/PLGA/PLA based NPs	PYT NPs	DSPE-mPEG	Terbinafine, Y8	Photo-immunotherapy	128	Tunable property, biocompatibility, prolonged circulation time, and low immunogenicity
	FA-PEG-S-S-PCL@PTX	FA-PEG-S-S-PCL	Paclitaxel	Chemotherapy	129	
	MiR-200/omSLN-CMN and Iri/omLip-CMN	PEG	Liposome, SLN, irinotecan, miR-200	Chemotherapy, gene therapy	130	
	ATRA-PLGA-PEG-PD-L1 NPs	PLGA-PEG	Antitumor agent all-trans retinoic acid (ATRA), anti-PD-L1 antibody	Immunotherapy	131	
	Cet-PEG-PLA-5Fu-^131^ I	PEG-PLA	Cetuximab, 5-FU, radionuclide iodine-131	Radiotherapy, chemotherapy	133	
	N/A	PEG-PLA-PEG	Cetuximab, cisplatin, 5-FU	Chemotherapy	134	

### 4.3 Metallic NPs

**Fig. 5:**
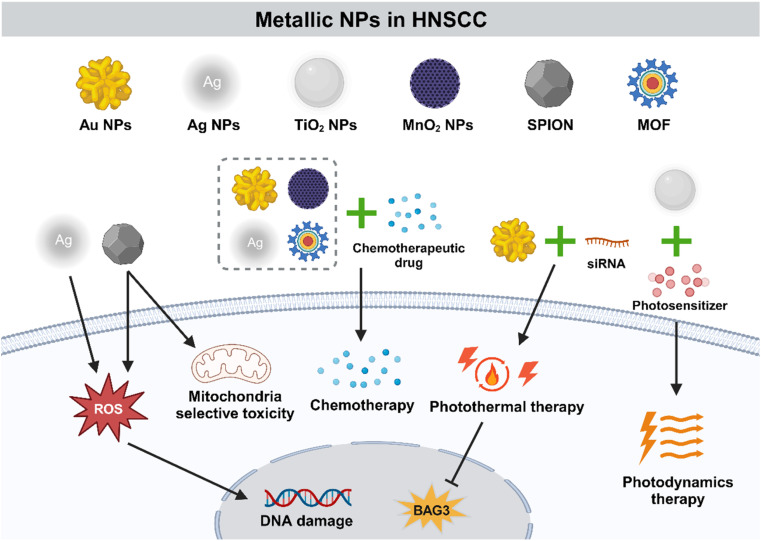
Metallic NPs are particularly advantageous nanomedicine in the treatment of OSCC. In addition to the direct tumor cell killing effect, metallic NPs play an important role in PDT and PTT owing to their superior photothermal properties. Accurately controlled physical characteristics make metallic NPs potential candidates for HNSCC therapy.

Metallic NPs (MNPs) are especially advantageous in oncotherapy as their shape, size, charge, and surface modification could be accurately controlled (Fig. 5). In addition, they are more easily internalized by cells than non-metallic NPs with the same size. Pure MNPs mainly contain those of gold (Au) and silver (Ag), while titanium dioxide, zinc oxide, and iron oxide are metal oxide NPs, which exhibit different physical and biological properties.^135^

#### 4.3.1 Au NPs

Au NPs are attractive for excellent stability, superior biocompatibility, and flexible tailorable morphology. A previous study has demonstrated that Au NPs exhibited cancer-associated-fibroblasts (CAF) and tumor inhibition potential *in vivo*.^136^ Au NPs are used to precisely deliver chemotherapeutic agents for negligible toxicity, easy modifying surface, and high drug-loading capability.^137^ Khamaikawin *et al.* encapsulated a triple chemotherapy drug formulation including docetaxel, cisplatin, and 5-FU into Au NPs, which enhanced the internalization efficiency and cytotoxicity *in vitro*, and exhibited a controlled drug-release profile at 24 h.^138^

Au NPs have been proved to be good candidates for PTT due to various optical properties such as localized surface plasmon resonance property, surface-enhanced Raman scattering (SERS) property and photothermal property with good biocompatibility and easy synthesis.^139–142^ Gold nanorods (GNRs), a type of capsule-like NP, have a pseudo-one-dimensional structure and size ranging in nanometers. Wang *et al.* developed GNRs-siRNA by binding siRNA to GNRs and significantly enhanced the PTT efficacy by targeting cancer cells and blocking the gene BAG3 of heat shock response.^143^ Moreover, the combination of Au NPs and appropriate antibodies could obtain more accurate tumor targeting. For instance, Au NPs have become selective agents for targeted PTT of HNSCC cells by conjugating to anti-EGFR antibodies.^144^

#### 4.3.2 Ag NPs

Ag NPs are important nanotechnology materials with many applications in different areas such as medicine, biology, and chemistry. Ag NPs showed great promise for oncotherapy *via* ROS production, which conducted specific toxicity to tumor cells through DNA damage with biocompatibility in other tissues.^145^ Additionally, the fabrication of nanocomposites provides an opportunity to improve the property of Ag NPs. Yang *et al.* prepared a chemotherapeutic nanoformulation: *M. chamomilla* aqueous extract-loaded Ag NPs. Ag NPs could reduce cell viability in HSC-4, Ca9-22, and HSC-3 cell lines in a dose-dependent manner, with minimized impairment to the non-tumor cells.^146^ In Satapathy's study, a PLGA-based uniform hybrid NP (QAgNP) containing bioactive small molecule quinacrine (QC) and Ag was prepared. The QAgNP showed higher cytotoxicity in H-357 oral cancer cells and OSCC stem cells than normal epithelial cells compared to QC and Ag NPs.^147^ Moreover, Xiong *et al.* prepared an αPDPN-Ag_2_S probe by decorating a podoplanin (PDPN) antibody on the surface of NIR-II Ag_2_S quantum dots (QDs), and the results revealed that the αPDPN-Ag_2_S probe had a tumor-targeting ability and therapeutic efficacy of eliminating partial epithelial–mesenchymal transition (pEMT) with acceptable biological safety. The αPDPN-Ag_2_S probe-conducted photoimmunotherapy remarkably changed the local immunosuppressive TME and improved PD-1 immunotherapy effectiveness.^148^

#### 4.3.3 Metal-oxide NPs

Metallic NPs could form a diversity of oxide compounds which lead to the formation of metal oxide NPs (MO-NPs). Titanium dioxide (TiO_2_) NPs are n-type semiconductor materials characterized by a refractive index in both the ultraviolet and visible bands, which make them widely used in optical techniques. Zhou *et al.* constructed TiO_2_@Ru@siRNA NPs consisting of ruthenium-based photosensitizer (Ru)-modified TiO_2_ NPs loaded with hypoxia-inducible factor-1α (HIF-1α)-siRNA. Under visible light irradiation, TiO_2_@Ru@siRNA NPs could elicit photodynamic effects, which significantly caused lysosomal damage, HIF-1α gene silencing, and HNSCC cell elimination. *In vivo* experimental results proved that TiO_2_@Ru@siRNA-mediated PDT greatly suppressed tumor progression and regulated tumor immunity through the adaptation to hypoxia.^149^ Nanostructured manganese dioxide (MnO_2_) NPs are a type of multifunctional tumor-therapeutic agents due to flexible structures, unique physicochemical property, and pH-responsive degradability. Hollow MnO_2_ (H-MnO_2_) nanostructures with mesoporous shells have been reported to be superior drug loading/delivery systems because of good load capacity and precise controlled release. Zhou and colleagues synthesized an intelligent platform based on H-MnO_2_ nanoshells that encapsulated chemotherapeutic drugs docetaxel and cisplatin (TP). The nanoshells were designed to alleviate tumor hypoxia, inhibit pathological angiogenesis, promote the dissolution of Mn^2+^, and synergize the impact of chemotherapy.^150^ Iron oxide NPs, which belong to the ferrimagnetic class of magnetic materials, are applied in the field of biomedical and bioengineering. Superparamagnetic iron oxide NPs (SPION) can be surface-modified and designed to different sizes and shapes. SPION are capable to conveniently cross the nuclear membrane and generate ROS, causing serious DNA damage to tumor cells.^151^ Additionally, Afrasiabi *et al.* evaluated the impact of SPION on HNSCC mitochondria. The results represented that SPION induced selective toxicity only in the mitochondria in HNSCC cells, demonstrating that SPION could be considered as a potential therapeutic candidate for HNSCC treatment.^152^ Furthermore, magnetic hyperthermia provides an alternative therapy by inducing thermal ablation of tumor tissues due to the heating property of magnetic NPs.^153^ Legge *et al.* successfully developed biocompatible silica-coated magnetic iron oxide NPs combined with antibodies targeting αVβ6 integrin to improve the killing effect on the targeted tumor cells in thermotherapy.^153^

#### 4.3.4 Metal–organic framework NPs

Metal–organic frameworks (MOFs) are self-assembly products of metal ions and organic ligands that have been recognized as attractive drug delivery nanoplatforms for oncotherapy due to their large surface area, good stability, tunable size and easy functionalization.^154,155^ Udesh Dhawan *et al.* synthesized FeAu alloy NPs (FeAu NPs), coated it with mesoporous iron(iii) trimesate (MIL-100) MOFs and encapsulated DOX within the nanostructures to fabricate FeAu@MOF NPs. The multimodal platform FeAu@MIL-100(Fe) eradicated oral cancer growth with excellent biocompatibility and bioimaging function.^156^ In addition, a type of HNSCC-targeted NP was obtained using dental pulp mesenchymal stem cell (DPSC) membranes to modify NPs of MOFs. The MOF@DPSCM complex conjugated with DOX has been identified to possess HNSCC-specificity and suppress CAL-27 tumor growth.^157^

As mentioned above, metallic NPs promote HNSCC inhibition and regression through various work mechanisms (Table 3 and Fig. 5). The specific physical and chemical characteristics of metallic NPs make them one of the most widely studied nanomaterials. With the deepening of research on the application of metallic NPs in the field of nanomedicine, the prospect of metallic NPs as an alternative selection for oncotherapy is inestimable.

**Table 3:** Metallic NPs applied for the treatment of head and neck squamous cell carcinoma

	Nanoparticles	Matrix	Agents	Biological applications	Ref.	Advantages
Au NPs	Docetaxel-cisplatin-fluorouracil-gold complex	Au NPs	Docetaxel, cisplatin, 5-FU	Chemotherapy	138	Direct antitumor effect, great stability, biocompatibility, flexible shapes, easy synthesis, high loading capability, and photothermal property
	GNRs-siRNA	Au NPs	BAG3-siRNA	Photothermal therapy, gene therapy	143	
	Anti-EGFR antibody conjugated gold NPs	Au NPs	Anti-EGFR antibody	Photothermal therapy	144	
Ag NPs	*M. chamomilla* aqueous extract containing Ag NPs	Ag NPs	*M. chamomilla* aqueous extract	Chemotherapy	146	Direct antitumor effect, easy modification, and biocompatibility
	QAgNP	PLGA-Ag hybrid NPs	Quinacrine (QC)	Chemotherapy	147	
	αPDPN-Ag_2_S	Ag_2_S quantum dots	Anti-podoplanin antibody	Photo-immunotherapy	148	
Metal oxide NPs	TiO_2_@Ru@siRNA	TiO2 NPs	Ruthenium-based photosensitizer (Ru), HIF-1α-siRNA	Photodynamic therapy, immunotherapy	149	Tuneable structures, unique physicochemical property, pH-responsive degradability, high loading capacity, and controlled-release property
	H-MnO_2_-PEG/TP nanoshells	Hollow MnO2 nanoshell	Docetaxel, cisplatin	Chemotherapy	150	
	SPIONs	Superparamagnetic iron oxide NPs (SPION)	N/A	Targeted therapy	152	
	Anti-αvβ6-conjugated MNP	Silica-coated magnetic iron oxide NPs	Anti-αVβ6 integrin antibody	Thermotherapy	153	
Metal–organic frameworks NPs	FeAu@MIL-100(Fe)	MIL-100(Fe)-coated FeAu alloy MOFs	Doxorubicin (DOX)	Chemotherapy, bioimaging	156	Large surface area, high stability, tunable size, and easy modification
	MOF@DPSCM	Dental pulp mesenchymal stem cell (DPSC) membranes-modified MOFs	DOX	Chemotherapy	157	

### 4.4 Non-metallic inorganic NPs

**Fig. 6:**
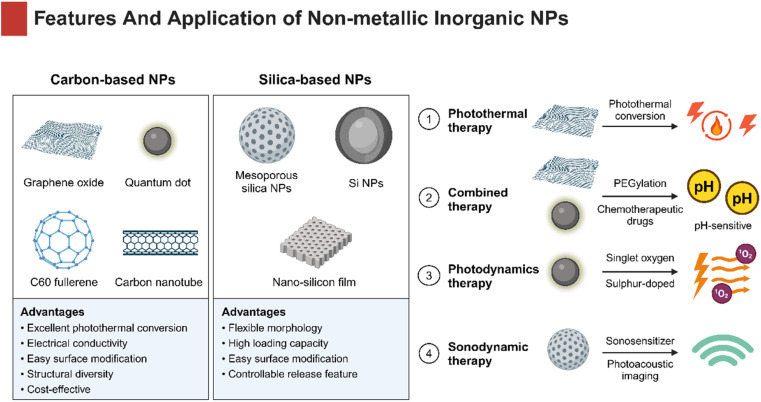
Non-metallic inorganic NPs have shown vast perspectives for oncotherapy. Flexible-changed shape, size, and surface morphology make them suitable carriers for drug delivery. Non-metallic inorganic NPs serve as a versatile platform to provide various and combined antitumor effects in HNSCC treatment.

Non-metallic inorganic NPs have been broadly applied as drug delivery systems in oncotherapy. Carbon and silicon are the two most abundant elements on the earth, making them the most commonly used non-metallic substances. These materials have an appropriate hardness and provide advantages of low cost and high biocompatibility, being ideally suitable for medical therapeutics (Fig. 6).

#### 4.4.1 Carbon-based NPs

Carbon-based NPs are endowed with plenty of superiorities such as electrical conductivity, high strength, outstanding surface chemistry, and wide range of structural diversity. Among them, nano-graphene oxide (NGO) and carbon dots (CDs) have been studied most extensively, on account of cost-effective, stable, biodegradable, and excellent photothermal conversion, which makes them particularly suitable for PTT.

NGO has attracted much attention for large specific surface area, great drug-entrapment capacity, pH-responsiveness, and EPR effect based on a two-dimensional crystal structure. A type of frequent and useful modification of NGO includes PEG functionalization, which significantly increase the stability in an aqueous solution and reduce nonspecific absorption to biological molecules and cells.^158^ For example, Li *et al.* designed a NGO-PEG hybrid delivery system to load DOX, which showed significantly higher cytotoxicity and considerable tumor-targeting in HNSCC cells compared to free DOX.^158^ Similarly, a gastrin-releasing peptide receptor (GRPR)-targeted NGO nanoprobe was successfully developed to realize pH-sensitive drug release and cytotoxicity in HSC-3 cells.^159^ Moreover, NGO has been verified useful in PTT for OSCC. Li *et al.* constructed fibroblast activation protein (FAP)-targeting PEGylated-NCO to encapsulate DOX and it called NPF@DOX. Due to its excellent photothermal conversion ability, the NPF@DOX showed superior thermogenic effect that greatly induced DOX-release and pH-stimulated cell apoptosis.^160^

In addition to NGO, CDs are equally promising for established size and surface functionalization, superior optical and biological properties.^161^ Appropriate size of CDs allows adjunctive cargoes to easily pass through the nuclear pore and stay in cells, helping reverse hypoxia-induced drug efflux and chemoresistance. Recent research studies revealed that graphene quantum dots (GQDs) were suitable for drug delivery. For instance, Wei *et al.* developed a tumor-targeted PEGylated-GQDs-cisplatin nanocomposite (GPt), resulting in improved chemotherapeutic efficacy for HNSCC.^162^ CDs are also playing a significant role in the fields of bioimaging and biosensing as fluorescent carbon NPs.^163,164^ Li *et al.* synthesized sulphur-doped carbon dots (S-CDs) as photosensitizers for PDT, which could automatically enter tumor cells and induce high-yield singlet oxygen by light, indicating better tumor-killing effects compared with the classic photosensitizer 5-ALA.^165^ Zhang *et al.* prepared manganese-doped carbon dot (Mn-CD) nanoenzymes that could lead to the decomposition of acidic H_2_O_2_*in situ* to generate O_2_. The successful development of Mn-CDs not only enhanced the PDT effect against HNSCC cells, but also witnessed the advances of water-soluble photosensitizers with oxygen production, demonstrating the superiority of PDT applied in hypoxic tumors.^166^

#### 4.4.2 Silica (Si)-based NPs

Si NPs have played a crucial role in plenty of fields including food industry, synthetic processes, medical diagnosis, and drug delivery. Among them, mesoporous silicon dioxide NPs (MSNPs) are especially valued for outstanding physicochemical properties of flexible morphology, high loading capacity, easy surface functionalization, biocompatibility and biodegradation. These characteristics allow MSNPs to have a high payload of antitumor agents and controlled-release ability. Based on superior delivering performance of MSNPs, Wang *et al.* employed polyethylenimine (PEI)-modified MSNPs to carry both multiple drug resistance protein 1 (MDR1)-siRNA and DOX. The MSNP-based complex significantly improved chemotherapeutic effectiveness in terms of treating multidrug-resistant cancer compared to free DOX.^167^ Another study has also reported that hyaluronic acid (HA)-assembled MSNPs acting as drug nanocarriers could precisely control drug release and internalization in CAL-27 cancer cells, suggesting that the HA-MSNPs-based vehicle is a promising platform for HNSCC treatment.^168^

MSNPs are used to establish a multifunctional nanotherapeutic platform, enhancing the efficacy of combination therapy for HNSCC. The AE105 (urokinase plasminogen activator receptor (uPAR)-targeting ligand)-decorated dendritic MSNPs entrapping photonic active ultrasmall Cu_2−*x*_S NPs and sonosensitizer Rose Bengal have been successfully synthesized and named CRDA. The CRDA, desired for its high biocompatibility, has achieved HNSCC-targeted synergistic therapeutic effects in the tumor model.^169^ In addition, Zhang and co-workers designed an HA-decorated Au–Si complex multifunctional drug delivery system loaded with DOX. Interestingly, the system successfully accomplished the photoacoustic imaging (PAI)-guided chemo-photothermal combined therapy, which exhibited a better antitumor capacity than chemotherapy or PTT alone.^170^

For summary, carbon-based NPs and silica-based NPs could maintain a stable size and geometry in biological applications due to ordered structures. Moreover, they are able to meet multiple needs *via* simple surface modifications. Therefore, non-metallic inorganic NPs are important therapeutic agents for HNSCC (Table 4 and Fig. 6).

**Table 4:** Non-metallic inorganic NPs applied for the treatment of head and neck squamous cell carcinoma

	Nanoparticles	Matrix	Agents	Biological applications	Ref.	Advantages
Carbon based NPs	DOX@NGO-PEG-HN-1	PEGylated NGO	Doxorubicin (DOX), OSCC cells-targeted peptide (HN-1)	Chemotherapy, targeted therapy	158	Electrical conductivity, high strength, excellent surface chemistry, flexible structure, cost-effective, great stability, biodegradable, and photothermal property
	DOX@NGO-BBN-AF750	NGO	DOX, gastrin-releasing peptide receptor (GRPR)-targeted bombesin antagonist peptide	Chemotherapy, targeted therapy	159	
	NPF@DOX	PEGylated NGO	DOX, fibroblast activation protein (FAP)-targeted peptide	Chemotherapy, photothermal therapy	160	
	GPt	PEGylated GQDs	Cisplatin	Chemotherapy	162	
	S-CDs	Sulphur-doped CDs	N/A	Photodynamic therapy	165	
	Mn-CDs	Manganese-doped CDs	N/A	Photodynamic therapy	166	
Silica-based NPs	MSNP-PEI-DOX/MDR1-siRNA	Polyethylenimine (PEI)-modified MSNPs	Multiple drug resistance protein 1 (MDR1)-siRNA, DOX	Chemotherapy	167	Flexible morphology, high loading capacity, easy surface functionalization, biocompatibility, biodegradation, and controlled-release property
	HA-siTMSN platform	Hyaluronic acid (HA)-assembled MSNPs	MTH1 inhibitor TH287, MDR1-siRNA	Chemotherapy, gene therapy	168	
	Cu_2−*x*_S-RB@DMSN-AE105	Dendritic MSNPs	uPAR-targeting ligand AE105, ultrasmall Cu_2−*x*_S NPs, Rose Bengal (RB)	Photothermal therapy, sonodynamic therapy	169	
	DOX-AuNRs@mSiO_2_-HA	Hyaluronic acid (HA) modified gold nanorods, MSNPs	DOX	Chemo-photothermal combined therapy	170	

## 5. Discussion

As the two most commonly used clinical treatment methods, a large number of studies on NPs for HNSCC have focused on radiotherapy and chemotherapy. The effect of NPs on radiotherapy is mainly reflected in enhancing the lethality of radiation to tumor cells or reducing the damage of normal tissues. For example, metallic NPs may enhance the tumor killing effect by absorbing ray energy and converting it into thermal energy. Organic NPs are chosen to be coated with radiation sensitizers to enhance their concentration in the tumor site. In chemotherapy, lipid-based NPs improve the efficiency of drug delivery by enhancing the interaction with cell membranes. By adjusting the chain length and crosslinking degree of polymer, polymeric NPs are able to achieve controlled-release and long-term therapy. In addition to radiotherapy and chemotherapy, NPs could contribute to the therapeutic effectiveness of other various treatment methods. For instance, NPs impacted angiogenesis and immune cell infiltration through the regulation of various factors in TME, inhibiting tumor growth and metastasis. Moreover, NPs with outstanding optical properties such as carbon nanotubes, quantum dots, and metallic NPs play an important role in PTT and PDT by excellent photothermal conversion. Briefly, most NPs act as carriers to deliver therapeutic molecules to tumor tissues, which could also achieve stimuli-triggered and precise tumor killing with material modifications. Only a few types of NPs such as metallic and carbon-based NPs possess intrinsic anti-tumor properties due to the characteristics of their particle composition. That is to say, the mechanism of anti-tumor activity of NPs *per se* is actually not clear, and whether they bring potential safety concerns is also worthy of discussion. More studies on the interaction between NPs and tumor mechanisms will significantly assist the application of NPs in tumor treatment.

Additionally, as the most common type of HNSCC, the alternative therapies for OSCC have been widely studied and differed from others in HNSCC. Considering the occurrence location of OSCC characterized with the commensalism of various oral microorganisms, it is worth mentioning that nanomedicine represented by extracted bacterial membranes and bacterial outer membrane vesicles is becoming a new trend of antitumor bacterial therapy.^171,172^ The advantages of bacteria-derived NPs can be summarized in several aspects: (i) colonizing various human tissues, especially tumor tissues; (ii) promoting microflora balance for close relationship with unique oral microenvironment; (iii) stimulating macrophage activation and modulating the immunosuppressive TME; (iv) maintaining more reliable biosafety *via* numerous approaches such as attenuation and inactivation; (v) constructing multifunctional nanoplatforms depending on easy transfection and surface modification.^173–176^ Therefore, there is no doubt that bacteria-derived NPs are emerging as a new option for nanotherapy at a surprising rate. In addition to bacteria-derived NPs, some other NPs have been designed to modulate oral microbiota through delivering inhibitory components for specific flora, which could activate antitumor immune response.^173^ These studies repeatedly demonstrated the promising prospects of bacteria therapy for OSCC.

**Fig. 7:**
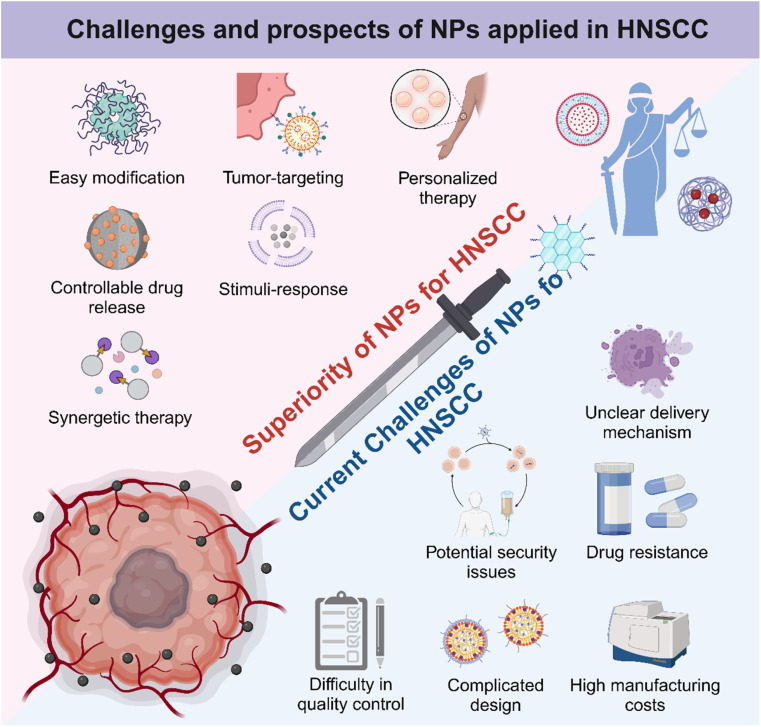
Current challenges and future prospects of NPs applied in HNSCC therapy. The application of NPs could work like a double-edged sword. NPs have exhibited irreplaceable advantages in drug delivery and synergetic therapy, greatly improving the antitumor effectiveness, while hidden security risks, high-cost and complicated preparation have caused widespread concern about nanotherapy. Therefore, how to use this “double-edged sword” to find the future directions of HNSCC treatment has a long way to go.

In summary, as Fig. 7 illustrates, NPs have unique advantages and potential in the treatment of HNSCC. By analyzing their structural properties, researchers can make better use of these NPs to design and develop efficient and low-toxicity tumor therapies. Due to high cargo loading capacity and easy structural editing, NPs have become powerful candidates for combined therapy and personalized treatment. Compared to monotherapy, multifunctional nanomedicine based on NPs exhibited synergetic tumor kill effectiveness, contributing to shortened treatment duration and improved prognosis.^177–179^ Moreover, HNSCC occurs in shallow mucosa and skin, and the pathological tissues are always visible. Therefore, topical administration of nanomedicine possesses promising clinical applications, and the abilities of local retention, cargo release, and mucosal and skin penetration should also be mainly considered in the next-generation nanotechnology-based approaches for HNSCC, which may be much more convenient and safer than systemic administration. All in all, there are several development strategies to help NPs maximize their efficacy and minimize side effects in HNSCC therapy. First, considering the EPR effect that renders NPs with a unique tumor aggregation ability, developing enhanced tumor targeting NPs continued to help achieve precise tumor killing. Further, by combining with some non-invasive imaging or diagnostic techniques, dynamic tracer anti-tumor NPs can be developed, which can greatly reduce the damage to normal tissue.^180^ In addition, the development of nanomaterials with local adhesion is very suitable for the treatment of HNSCC.

Despite significant advancements in the utilization of NPs for HNSCC treatment, it has become evident that the NPs are increasingly intricate-designed, often incorporating multiple functional components to meet complicated needs. This trend raises concerns regarding the escalating complexity in elucidating their synergistic mechanisms of action. As mentioned earlier, more research studies are needed to confirm the mechanism of the anti-tumor effects of NPs. Additionally, the costs and complexities associated with NP preparation must be carefully considered. The balance of cost, manufacturing difficulty, and functionality of NPs requires more practice to evaluate and achieve. Moreover, the safety and toxicity of NPs need to be further studied due to the lack of follow-up observations, toxicological experiments, and proper tumor models. Therefore, developing humanized animal models with true humanized tumor characteristics would greatly help reduce heterogeneity and promote the clinical transformation efficiency of anti-tumor nanomaterials by extending the observation time and increasing observation indicators.^181,182^ Besides, emerging biomimetic NPs including EXOs, cell membranes, and bacteria-derived NPs may lead to unexpected security issues for potential biological regulatory abilities. Some studies have reported difficulty in the quality control of biomimetic products containing biological ingredients, while others have expressed concern about the lack of a fully clear transporting mechanism for biological-origin NPs.^87,183^ Developing cell-based biomimetic NPs is also controversial for tumorigenic potential and ethical issues.^184,185^ Therefore, it is necessary to conduct a standardized and highly repeatable procedure for the extraction, purification and characterization of biomimetic NPs. Moreover, developing personalized medicine approaches like the use of patient-derived organoids would be valuable to evaluate the efficacy and safety of biological-origin NPs in a patient-specific manner.^186^ This kind of personalized treatment strategy would enhance therapeutic outcomes and decrease treatment-related toxicity. Briefly, the challenges of scaling up production, controlling costs, and ensuring long-term safety need to be addressed urgently to fully harness the potential of nanotechnology in oncotherapy (Fig. 7). Nevertheless, the future remains promising for the application of NPs in HNSCC treatment, and further advancements in this exciting field are worth looking forward to.

## 6. Conclusion

HNSCC is a type of malignant tumor with high incidence and poor prognosis. Classic therapeutic approaches show numerous shortcomings, and thus, limit the therapeutic effect. NPs with unique physicochemical properties have demonstrated promising potential in HNSCC therapy. These NPs could effectively deliver therapeutic agents to tumor sites, enhancing antitumor activity with minimal side effects. Advances in nanotechnology have enabled the development of targeted drug delivery systems, which can precisely locate and destroy tumor cells while sparing healthy tissues. NPs designed to respond to specific biological markers or environmental stimuli make precise treatment a reality. Furthermore, multifunctional nanotherapy platforms have been successfully developed and demonstrated efficacy, shedding new light on the combination therapy for HNSCC. With more research studies focusing on the safety and long-term effects of NPs in patients, nanomedicine shows great potential to open a new chapter for oncotherapy.

## Data availability

Data are available from the authors upon request.

## Author contributions

Bicai Tang contributed to the writing – review and editing and validation of the manuscript. Rui Huang draft the manuscript. Wenjuan Ma contributed to the conceptualization, validation and supervision of the manuscript.

## Conflicts of interest

All authors declare no conflicts of interest.
